# 

**Published:** 1882-03

**Authors:** 


### CONTENTS.

COMMUNICATIONS.

Anæsthetics Medico-Legally Considered.......... 103

SELECTIONS.

Some Hints on the Preparation and Mounting of Microscopic Objects, 123
Ammonia—Its Relations to Dentistry.......... 133
A Case in Practice.......... 142
Biographical—Dr. M. S. Dean.......... 145
Fistula Beneath the Chin, of Four Year's Standing.......... 148
Earache.......... 149

EDITORIAL.

The Freezing Cure.......... 150

Transactions.......... 151

In Memoriam.......... 151

Michigan Dental Association.......... 152

Biographical.......... 153

Just Closed.......... 154

